# A common symptom geometry of mood improvement under sertraline and placebo associated with distinct neural patterns

**DOI:** 10.1017/S0033291725100962

**Published:** 2025-07-04

**Authors:** Lucie Berkovitch, Kangjoo Lee, Jie Ji, Markus Helmer, Masih Rahmati, Jure Demsar, Aleksij Kraljic, Andraz Matkovic, Zailyn Tamayo, John Murray, Grega Repovs, John Krystal, William Martin, Clara Fonteneau, Alan Anticevic

**Affiliations:** 1 Department of Psychiatry, Neuroscience, and Psychology, Yale University School of Medicine, New Haven, CT, USA; 2 Division of Neurocognition, Neurocomputation, Neurogenetics (N3), Yale University School of Medicine, New Haven, CT, USA; 3 Université Paris Cité, Paris, France; 4 Department of Psychiatry, GHU Paris, Psychiatrie et Neurosciences, Service Hospitalo-Universitaire, Paris, France; 5 Institut de Neuromodulation, GHU Paris, Psychiatrie et Neurosciences, Centre Hospitalier Sainte-Anne, Pôle Hospitalo-universitaire, Université Paris Cité, Paris, France; 6 Unicog, Saclay CEA Centre, Neurospin, Gif-Sur-Yvette Cedex, France; 7 Department of Psychiatry, Yale University School of Medicine, New Haven, CT, USA; 8 Department of Psychology, University of Ljubljana, Ljubljana, Slovenia; 9 Faculty of Computer and Information Science, University of Ljubljana, Ljubljana, Slovenia; 10 Department of Psychological and Brain Science, Dartmouth College, Hanover, NH, USA

**Keywords:** data reduction, functional neuroimaging, mood spectrum, statistical learning, symptoms mapping personalized patient selection

## Abstract

**Background:**

Understanding the mechanisms of major depressive disorder (MDD) improvement is a key challenge to determining effective personalized treatments.

**Methods:**

To identify a data-driven pattern of clinical improvement in MDD and to quantify neural-to-symptom relationships according to antidepressant treatment, we performed a secondary analysis of the publicly available dataset EMBARC (Establishing Moderators and Biosignatures of Antidepressant Response in Clinical Care). In EMBARC, participants with MDD were treated either by sertraline or placebo for 8 weeks (Stage 1), and then switched to bupropion according to clinical response (Stage 2). We computed a univariate measure of clinical improvement through a principal component (PC) analysis on the variations of individual items of four clinical scales measuring depression, anxiety, suicidal ideas, and manic-like symptoms. We then investigated how initial clinical and neural factors predicted this measure during Stage 1 by running a linear model for each brain parcel’s resting-state global brain connectivity (GBC) with individual improvement scores during Stage 1.

**Results:**

The first PC (PC1) was similar across treatment groups at stages 1 and 2, suggesting a shared pattern of symptom improvement. PC1 patients’ scores significantly differed according to treatment, whereas no difference in response was evidenced between groups with the Clinical Global Impressions Scale. Baseline GBC correlated with Stage 1 PC1 scores in the sertraline but not in the placebo group.

Using data-driven reduction of symptom scales, we identified a common profile of symptom improvement with distinct intensity between sertraline and placebo.

**Conclusions:**

Mapping from data-driven symptom improvement onto neural circuits revealed treatment-responsive neural profiles that may aid in optimal patient selection for future trials.

## Introduction

Major depressive disorder (MDD) is a frequent and heterogeneous psychiatric disease (World Health Organization, 2017). The use of a traditional categorical approach derived from the Diagnostic and Statistical Manual of Mental Disorders (5th edition) criteria (American Psychiatric Association, 2013) severely limits the identification of treatment response predictors (Perlman et al., 2019). Clinical trials traditionally use predefined scales to compare treatment with a placebo. However, an existing gap in our field relates to whether there could be a generalized response pattern that cuts across active treatment and placebo (Gueorguieva, Mallinckrodt, & Krystal, 2011; Huneke et al., 2022). Indeed, clinical improvement obtained after antidepressant treatment embeds a placebo response, and both antidepressants and placebos can induce neurobiological changes that may share commonalities (Benedetti et al., 2005). Furthermore, it remains unknown if patients’ baseline neural configurations are associated with a unique clinical response pattern. In this study, we intend to test whether a common data-driven profile of improvement can be identified across both treatment and placebo groups. We evaluated data from the publicly available dataset EMBARC (Establishing Moderators and Biosignatures of Antidepressant Response in Clinical Care). EMBARC is a multisite randomized placebo-controlled trial in which unmedicated participants with MDD were treated either by sertraline, a serotonin selective reuptake inhibitor, or placebo, and then switched to bupropion (an atypical antidepressant) according to clinical response status (Trivedi et al., 2016). EMBARC collected clinical measures (including item-level data on depression, anxiety, suicidal ideas, and manic-like symptoms scales) and baseline neuroimaging data to find markers associated with antidepressant treatment outcomes (Ang et al., 2022; Beliveau et al., 2022; Chin Fatt, Cooper, Jha, Aslan, et al., 2021; Chin Fatt, Cooper, Jha, Minhajuddin, et al., 2021; Chin Fatt et al., 2020, 2023; Cooper et al., 2019, 2019; Fan et al., 2020; Webb et al., 2019; Whitton et al., 2019; Zhao et al., 2023). One of the specific aspects of EMBARC is the remarkable intensity of the placebo effect. Using the Clinical Global Impressions (CGI) Scale, a clinician rated a nonspecific 7-point scale providing a single global measure of improvement, or global clinical scores such as the Hamilton Rating Scale for Depression (HRSD); no difference in clinical outcomes was evidenced between the placebo and the sertraline groups (Chin Fatt et al., 2020; Cooper et al., 2019; Fan et al., 2020; Pizzagalli et al., 2018; Webb et al., 2019; Whitton et al., 2019). However, clinical and neurological predicting factors of sertraline and placebo response were identified, some of them being shared (Fan et al., 2020; Pizzagalli et al., 2018) and others being specific to the sertraline or the placebo group (Ang et al., 2022; Chin Fatt et al., 2020, 2023; Chin Fatt, Cooper, Jha, Aslan, et al., 2021; Chin Fatt, Cooper, Jha, Minhajuddin, et al., 2021; Cooper et al., 2019; Webb et al., 2019).

Here, we applied a new analytic strategy to evaluate whether the placebo and the sertraline groups share a common pattern of symptom improvement. Specifically, we performed a principal component analysis (PCA) on the variations of individual items of four clinical scales, resulting in a univariate score of clinical improvement. We then compared the sertraline and placebo groups using this score and investigated whether clinical and neural factors at baseline could predict it. With this approach, we were able to identify a common clinical profile of symptom improvement that occurs across placebo and sertraline. Critically, however, both the intensity of response reflected by this score and the baseline neural patterns linked to symptom improvement showed a clear difference between the treatment and placebo groups.

## Materials and methods

### Data collection, study design, and clinical sample

**Figure 1.**
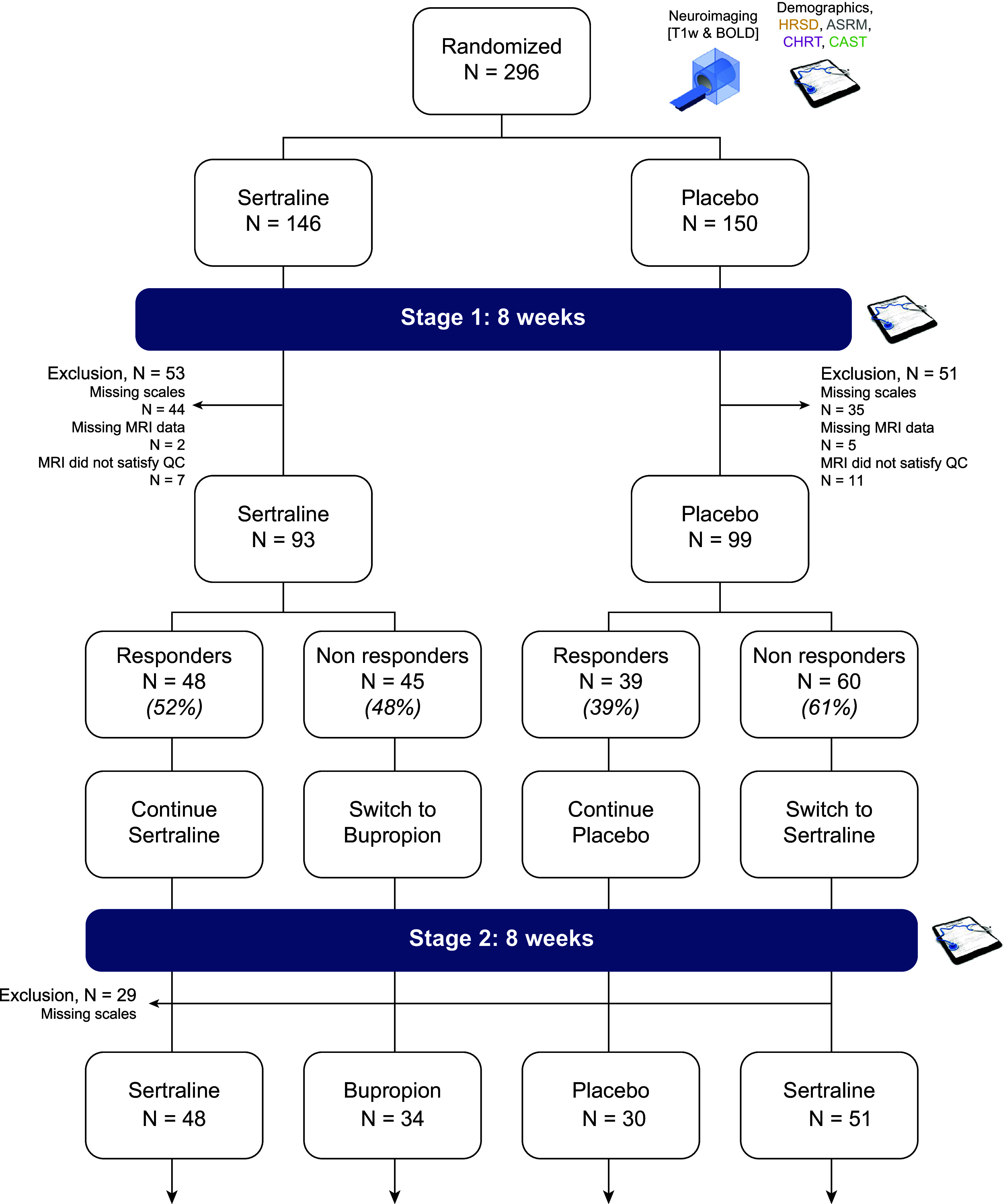
Study design and analyzed population. In Stage 1, patients with early-onset recurrent MDD were randomized to receive either sertraline up to 200 mg daily or placebo under double-blind conditions. At Week 8, participants were assessed with the CGI, and those who had a score of less than “much improved” were considered nonresponders. In Stage 2, nonresponding patients in Stage 1 were switched to another treatment under double-blind conditions: sertraline nonresponders received bupropion, and placebo nonresponders received sertraline. Responders continued the treatment received during Stage 1. Participants underwent an extensive clinical assessment and a 3T MRI at baseline. At 8 and 16 weeks, four clinical scales of interest were performed: (i) Hamilton Rating Scale for Depression (HRSD), which assesses depressive symptoms; (ii) Altman Self-Rating Mania Scale (ASRM), which measures manic symptoms; (iii) Concise Health Risk Tracking (CHRT), which evaluates suicidal propensity and risk; and (iv) Concise Associated Symptoms Tracking Scale (CAST), which reflects both anxiety, irritability, and manic symptoms.

In EMBARC, patients with MDD received either sertraline (up to 200 mg daily) or placebo for 8 weeks (Stage 1). Then, their treatment was adapted according to their CGI rating for 8 additional weeks under double-blind conditions (Stage 2), a CGI score of less than “much improved” at 8 weeks, being considered as a nonresponse (Trivedi et al., 2016) (see Figure 1). During Stage 2, sertraline nonresponders received bupropion, placebo nonresponders received sertraline, and responders continued the treatment they received during Stage 1. Participants underwent a magnetic resonance imaging (MRI) at baseline and an extensive clinical assessment at 8 and 16 weeks, including four clinical scales of interest: (i) HRSD (Hamilton, 1960), which assesses depressive symptoms; (ii) Altman Self-Rating Mania Scale (ASRM) (Altman, Hedeker, Peterson, & Davis, 1997), which measures manic symptoms; (iii) Concise Health Risk Tracking (CHRT) (Trivedi et al., 2011), which evaluates suicidal propensity and risk; and (iv) Concise Associated Symptoms Tracking Scale (CAST) (Trivedi et al., 2011), which reflects both anxiety, irritability, and manic symptoms. We included 192 patients who had full clinical data and quality-controlled neuroimaging data at baseline (see Figure 1 and Table 1).

**Table 1.** Baseline patient characteristics

Characteristics	PLA, *N* = 99^a^	SER, *N* = 93^a^	*p*-value^b^
Gender			0.5
Female	63 (64%)	64 (69%)	
Male	36 (36%)	29 (31%)	
Age (years)	37.73 (1.30)	37.73 (1.46)	>0.9
Ethnicity			0.7
American Indian/Alaska Native	1 (1.0%)	0 (0%)	
Asian	5 (5.1%)	5 (5.4%)	
Black or African American	16 (16%)	19 (20%)	
More than one race	4 (4.0%)	6 (6.5%)	
White	73 (74%)	63 (68%)	
Subject education (years)	15.39 (0.25)	14.95 (0.28)	0.2
Unknown	0	2	
Major depressive disorder: Severity at baseline			0.8
High	41 (41%)	41 (44%)	
Low	58 (59%)	52 (56%)	
Major depressive disorder: Chronicity at baseline			0.9
Chronic	50 (51%)	49 (53%)	
Non-chronic	49 (49%)	44 (47%)	
HRSD: Hamilton Rating Scale for Depression (total score)	18.42 (0.44)	18.73 (0.44)	0.6
ASRM: Altman Self-Rating Mania Scale (total score)	1.30 (0.18)	1.42 (0.18)	0.6
CHRT: Concise Health Risk Tracking Scale (propensity score)	26.96 (0.84)	26.88 (0.81)	>0.9
CHRT: Concise Health Risk Tracking Scale (risk score)	5.39 (0.23)	5.54 (0.26)	0.7
CAST: Concise Associated Symptoms Tracking Scale (total score)	29.69 (0.91)	30.39 (0.99)	0.6

a *n* (%); Mean (SEM).

b Pearson’s *χ* ^2^ test; Welch two-sample *t*-test.

### Dimension reduction of symptom improvement

To estimate data-driven patterns of symptom improvement, we ran a three-step analysis on the clinical variation, that is, the difference in score for each item before and after treatment across all 73 items of the HRSD, ASRM, CHRT, and CAST scales. We first performed dimension reduction of clinical measures using PCA separately for each group. Then, we evaluated principal component (PC) reproducibility using cross-validation. Finally, we compared PC loadings and scores across groups to identify shared/distinct axes of symptom improvement. This strategy is similar to that employed in previous publications of our team (Ji et al., 2021; Lee et al., 2024; Moujaes et al., 2024).

This data dimension reduction approach enables the detection of differences between groups in two ways. First, separate group PCA can yield PCs that differ in terms of geometry (i.e., loadings), meaning that the type of symptoms changing over time depends on the treatment group. Alternatively, we can run PCA across groups, yielding common PCs and show that groups have different score distributions for these PCs (i.e., different “intensity” of change on common axes of clinical variation), using post-hoc two-sample two-sided *t*-tests. Additional details can be found in the Supplementary Methods.

### Dimension reduction of baseline symptoms

To explore clinical predictive factors of improvement, we applied the same approach to baseline symptoms. We run a PCA across participants, regardless of the group, to check whether baseline-symptom PC loadings would resemble those of the PCs reflecting improvement (i.e., obtained from the difference of score for each item before and after treatment) and whether baseline-symptom PC scores (i.e., severity at baseline) would predict improvement PC scores (i.e., intensity of clinical response).

### Neural data reduction via functional brain-wide parcellation

Neuroimaging data were acquired at baseline during resting state using a 3T MRI (Greenberg et al., 2015). All scans were processed using the Quantitative Neuroimaging Environment & ToolboX (QuNex, https://qunex.yale.edu/) (Ji et al., 2023), which integrates the Human Connectome Project Pipelines (Glasser et al., 2013). The preprocessing pipeline is described in the Supplementary Methods. Neural data were examined at different levels: parcels (718 parcels), networks (12 networks) and whole brain (brain average) to assess whether local, functional, or global patterns of connectivity would be predictive of clinical improvement. Data were first parcellated following the Cole-Anticevic Brain Network Parcellation (CAB-NP) atlas (Glasser et al., 2016; Ji et al., 2019). Global brain connectivity (GBC), which correlates the time series of every voxel (or area) with every other voxel (or area), was then calculated for each subject at the parcel level to reduce the dimensionality of the neural feature space (Ji et al., 2019) (see Supplementary Methods for details about GBC calculation). For network-level analyses, we averaged GBC values across parcels belonging to each network – at the cortical and subcortical levels – derived from the CAB-NP atlas. Subcortical structures of interest were chosen based on previous findings in depression (Chen et al., 2018; Chin Fatt et al., 2023; Chin Fatt, Cooper, Jha, Aslan, et al., 2021; Godlewska et al., 2018; Karim et al., 2018; Korgaonkar, Goldstein-Piekarski, Fornito, & Williams, 2020; Li et al., 2023; Lu et al., 2020; Martens, Filippini, Harmer, & Godlewska, 2022; Rolle et al., 2020; Strege et al., 2023; Wu et al., 2016). For the brain average level analyses, we averaged GBC values across the 718 parcels.

### Mass univariate brain-behavior mapping

The interactions between clinical improvement and individual baseline GBC at the parcel-level were measured via a mass univariate regression procedure (Ji, Spronk, et al., 2019). The resulting maps correspond to the regression coefficients between patients’ clinical improvement and GBC in each parcel, across all 192 patients. The greater the magnitude of the coefficient for a given location, the stronger the statistical relationship between GBC and the clinical variation across patients during Stage 1. The significance of the maps was assessed using Permutation Analysis of Linear Models (PALM) (Winkler et al., 2014). We computed two-tailed Pearson’s correlation, ran 1,000 permutations, and applied family-wise error rate correction.

To explore whether GBC was statistically associated with first PC (PC1) measures, we conducted analyses of variances (ANOVAs) with GBC as a dependent variable, PC1 in interaction with treatment and networks or subcortical structures, and age, gender, and site as covariates. We accounted for repeated measures per participant using a within-subjects factor (networks) within error structure.

In case there was a significant interaction between PC1 and treatment, we then measured whether PC1 could be predicted by GBC, respectively, in the sertraline and placebo groups. We ran ANOVA with PC1 score as a dependent variable, and GBC interacting with network or subcortical structure separately for each treatment group, and across different spatial regions (e.g., per functional brain networks including both cortical and subcortical regions and per subcortical structures). Additionally, we included age, gender, and site as independent variables. If there was a significant effect involving network or subcortical structure, we studied the correlation between PC1 and GBC for each network (or subcortical structure) separately.

Finally, if there was a significant interaction between PC1 and network or subcortical structures in the main ANOVA, we explored whether GBC in a given network or subcortical structures would predict PC1 regardless of treatment. To do so, we ran an ANOVA separately for each network/substructure, with PC1 score as a dependent variable, and GBC interacting with treatment, and age, gender, and site as independent variables.

## Results

### Analysis of total scale scores

**Figure 2.**
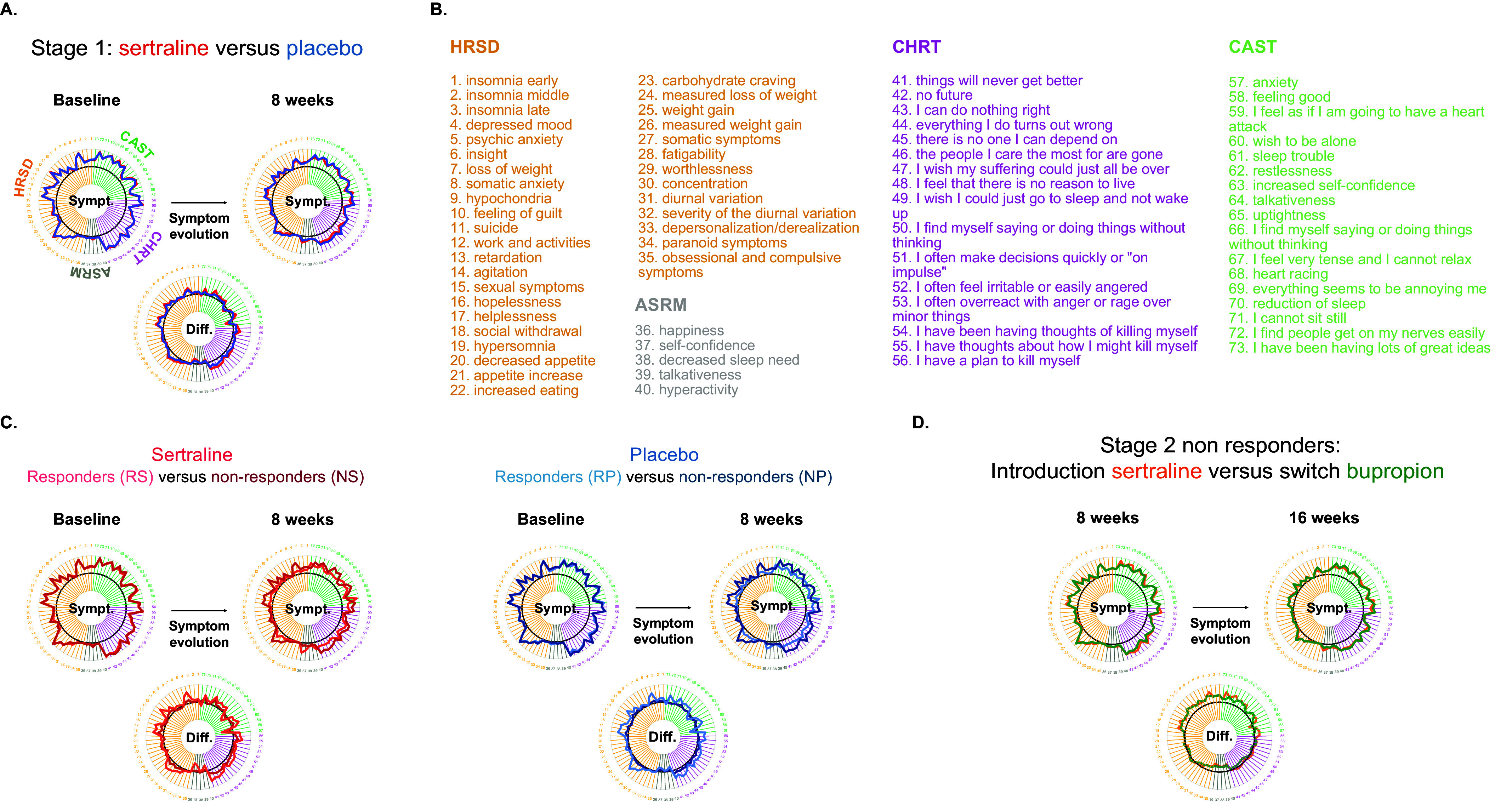
Symptom improvement during stages 1 and 2. (A) Symptom improvement during Stage 1 at the item level for each group (red: sertraline and blue: placebo): scores at the item level are similar between the two treatment groups at baseline and differ only for the CHRT scores evolution. (B) List of all 73 items across the four clinical scales. (C) Symptom improvement during Stage 1 in responders and nonresponders according to the CGI (salmon: responders to sertraline, brown: nonresponders to sertraline, light blue: responders to placebo, and dark blue: nonresponders to placebo). Responders and nonresponders have significant differences in symptom improvement for all clinical scales. (D) Symptom improvement during Stage 2 (green: patients switched from sertraline to bupropion, orange: patients switched from placebo to sertraline). Patients switched to bupropion have a lower global CHRT risk score compared to patients switched to sertraline at the beginning of Stage 2 and improved less than participants under sertraline during Stage 2.

We first looked at the total scale score variations for each stage. There was no difference at baseline between treatment groups (see Figure 2A). The CHRT was the only scale whose scores improved more in the sertraline than in the placebo group during Stage 1 (variation of CHRT propensity score: *p =* 0.009; CHRT risk score: *p =* 0.002). The proportion of responders and nonresponders according to the CGI was similar in the two groups at the end of Stage 1 (placebo 39.4% versus sertraline 51.6%, *χ* ^2^ = 2.4, *p =* 0.12). There were no significant differences at baseline between responders and nonresponders (all *p >* 0.08), suggesting that baseline symptoms were not predictive of subsequent CGI response status. A more detailed analysis of total scale scores at baseline and during each stage can be found in the Supplementary Results.

### Identifying clinical improvement geometry using PCA analysis

#### The geometry of the PC1 is consistent across groups

**Figure 3.**
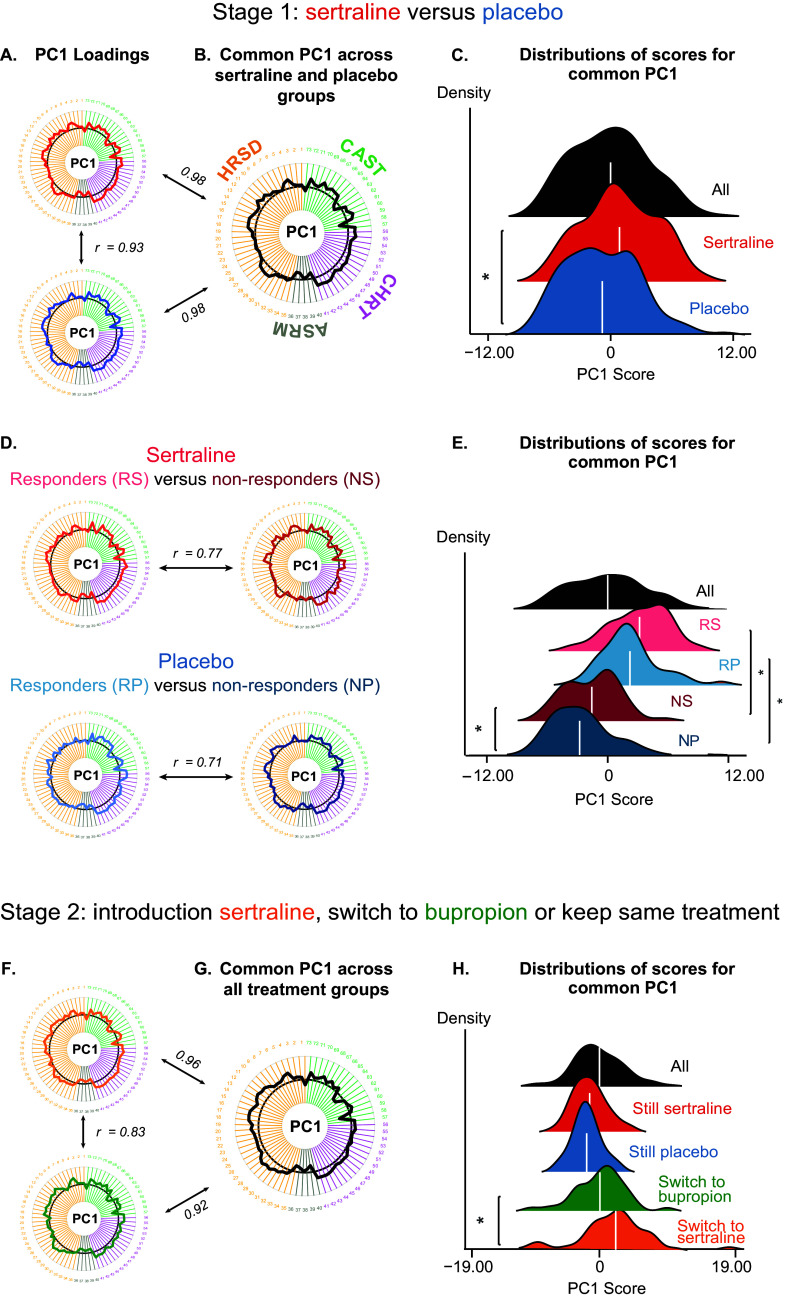
Principal component analysis on symptom improvement during stages 1 and 2. (A) PC1 loadings: Symptom improvement geometry is very similar between the two groups (red: sertraline and blue: placebo). (B) PCA pooling all participants yields another PC1 (common PC1: black), which is very similar to the PC1 resulting from PCA run separately on the two groups. (C) Distribution of scores for common PC1 in each group (red: sertraline subgroup, blue: placebo subgroup, and black: all participants). On average, patients under sertraline have higher scores than patients under placebo (*t*_190_ = 3.16, *p* = 0.0018). (D) Results of the same analyses but splitting each treatment group according to clinical CGI response status (salmon: responders to sertraline, brown: nonresponders to sertraline, light blue: responders to placebo, and dark blue: nonresponders to placebo). PCA again yields a PC1 that explains most variance and is reliable. (E) Distribution of scores for the PC1 run across all participants (common PC1) for each subgroup of treatment × CGI response. On average, patients not responding to sertraline have higher scores than patients not responding to placebo (*t*_97_ = 2.25, *p* = 0.027). (F) Results of the same analyses performed during Stage 2 (green: patients switched from sertraline to bupropion and orange: patients switched from placebo to sertraline). PCA again yields a PC1 that explains most variance is reliable and is relatively similar in terms of loadings between the two groups receiving a new medication. (G) Common PC1 loadings and score distribution for Stage 2. PCA pooling all participants yields another PC1 (common PC1: black), which is very similar to the PC1 resulting from PCA run separately in the different groups. (H) Distribution of scores for common PC1 in each group for Stage 2 (red: responders to sertraline during Stage 1, blue: responders to placebo during Stage 2, green: switched to bupropion, orange: switched to sertraline, and black: all participants). On average, patients who switched to sertraline have higher scores than patients who switched to bupropion (*t*_80_ = 2.39, *p* = 0.019).

To explore the geometry of symptom improvement across groups, we ran separate PCA on the clinical item evolution for each group during each stage: two groups – sertraline versus placebo – in Stage 1 (Figure 3A–E and Table 2), and four groups – sertraline responders, sertraline nonresponders switched to bupropion, placebo responders, and placebo nonresponders switched to sertraline – in Stage 2 (Figure 3F–H and Table 2). Reliability was assessed with split-half cross-validation. Except for responders during Stage 2, PCA yielded significant and reliable PC1 in all groups and stages. Other significant PCs were not reliable (*r*-values for split-half cross-validation below 50%, see Supplementary Figure S1) and were, therefore, not retained for further analyses. The geometry of PC1, explaining the most variance of symptom improvement in each group, was common across treatment groups, exhibiting high correlations of PC1 geometries between groups (Stage 1: placebo vs. sertraline, *r* = 0.93, *p* < 0.001; Stage 2: sertraline newly introduced vs. bupropion, *r* = 0.83, *p* < 0.001). Given this result, we ran an additional PCA on symptom improvement for each stage, pooling all the participants together (i.e., sertraline and placebo together for Stage 1 [Figure 3B] and all treatment groups together for Stage 2 [Figure 3G]). We confirmed that the resulting common PC1 estimated from all participants was highly correlated with the PC1s estimated from each group separately (see Figure 3B,G, Supplementary Results, and Supplementary Figure S2 for a detailed description of item loadings).

**Table 2.** Principal component analysis results

Group and stage	Number of significant PCs	Percentage of explained variance		PC1 reliability (split-half correlation)	Loadings correlation between PCs
		All PC	PC1		
Improvement stage 1					
Sertraline (*n* = 93)	6	42.6	19.2	0.87	0.93 *p <* 0.001
Placebo (*n* = 99)	5	38.1	19.7	0.86	
Sertraline-responders (*n* = 48)	3	42.6	14.5	0.56	0.78 *p <* 0.001
Sertraline nonresponders (*n* = 45)	6	38.1	19.7	0.60	
Placebo responders (*n* = 39)	5	38.5	15.8	0.56	0.71 *p <* 0.001
Placebo nonresponders (*n* = 60)	4	42.7	14.3	0.66	
Improvement stage 2					
Sertraline (previously placebo) (*n* = 60)	4	43.1	23.5	0.75	0.83 *p <* 0.001
Bupropion (previously sertraline) (*n* = 45)	4	43.2	19.2	0.56	
Sertraline responders (*n* = 48)	3	28.4	-	< 0.50	-
Placebo responders (*n* = 39)	8	66.3	-	< 0.50	
Improvement across all participants (common PC)					
Stage 1	7	42.6	19.6	0.93	0.95 *p <* 0.001
Stage 2	7	45.4	20.0	0.89	
Baseline PC					
Baseline	11	50.1	10.3	0.60	-

#### The sertraline group has higher PC1 scores than the placebo group, while responders have higher scores than nonresponders

The estimation of common PCs of symptom improvement allowed us to study the distributions of individual participants’ scores on a common axis of symptom improvement according to their group and to explore whether treatment groups had different score distributions on this common PC1.

During Stage 1, the sertraline group had, on average, higher common PC1 scores than the placebo group (*t*_190_ = 3.16, *p* = 0.0018, see Figure 3C). Responders also had higher scores than nonresponders (sertraline: responders vs. nonresponders: *t*_91_ = 8.23, *p* < 0.0001, placebo: responders vs. nonresponders: *t*_80_ = 8.57, *p* < 0.0001, Figure 3E). Nonresponders to sertraline had higher scores than nonresponders to placebo (*t*_97_ = 2.25, *p* = 0.027), whereas sertraline and placebo responders’ scores did not differ (*t*_82_ = 1.54, *p* = 0.13). During Stage 2, the sertraline group (formerly nonresponders to placebo in Stage 1) had a higher score than the bupropion group (formerly nonresponders to sertraline in Stage 1) (*t*_80_ = 2.39, *p* = 0.019, Figure 3H). However, it is worth noting that those two groups were defined on CGI response to placebo and sertraline, respectively. Therefore, they are not comparable in terms of treatment-resistance profiles (one group is naïve, whereas the other is sertraline-resistant) and have different sample sizes. The groups that received Stage 1 treatment did not significantly differ in their score distributions (*t*_80_ = 0.82, *p* = 0.42). Overall, using PC1, it appears that patients receiving sertraline have a higher clinical improvement than patients under placebo during Stage 1. Superiority of sertraline over bupropion is difficult to assert given differences in terms of resistance profile and sample size.

### Predicting clinical improvement

#### Clinical score at baseline can predict clinical improvement following treatment based on PC1 scores

We then evaluated whether individual clinical measures collected at baseline could predict individual treatment responses during Stage 1, using the principal axis of symptom improvement (common PC1 scores).

To do this, we estimated the correlation between Stage 1 PC1 scores and baseline clinical scores across subjects and performed an ANOVA test, where common PC1 scores are dependent variables and HRSD, ASRM, CHRT, and CAST baseline total scores and treatment groups (sertraline vs. placebo) are independent variables.

We found that CHRT risk and CAST global scores at baseline predicted Stage 1 PC1 scores across sertraline and placebo groups (CHRT: *r* = 0.20, *p =* 0.007; CAST: *r* = 0.20, *p =* 0.006) with no differences between the two groups (interaction CHRT × treatment: *F*_1,188_ = 0.6, *p =* 0.45; interaction CAST × treatment: *F*_1,188_ = 0.4, *p =* 0.52). In contrast, HRSD scores and depression severity at baseline did not predict Stage 1 PC1 scores across sertraline and placebo groups, but their interaction with the treatment group was significant (interaction HRSD × treatment: *F*_1,188_ = 5.8, *p =* 0.017; interaction depression severity × treatment: *F*_1,188_ = 5.6, *p =* 0.019). Indeed, HRSD scores and depression severity predicted Stage 1 PC1 scores in the sertraline group, but not in the placebo group (HRSD sertraline group: *r* = 0.21, *p =* 0.040 vs. placebo group: *r* = −0.13, *p =* 0.19; depression severity (high > low) sertraline group: *F*_1,91_ = 3.7, *p =* 0.058 vs. placebo group: *F*_1,91_ = 2.1, *p =* 0.15). Overall, these results suggest that suicidal risk (CHRT) and anxiety symptoms (CAST) at baseline predict clinical improvement as measured by PC1 scores, irrespective of treatment, whereas depression severity at baseline predicted sertraline efficacy specifically.

#### Principal axis of clinical items at baseline can predict clinical improvement following treatment

By running the PCA on the baseline symptoms as opposed to PCA on the delta between time points, we evaluated whether the baseline-symptoms principal axis of clinical scores could predict subsequent clinical improvement. The loadings of baseline-symptoms PC1 and common improvement PC1 during Stage 1 were found to be significantly correlated (*r* = 0.69, *p <* 0.001), indicating that the improvement concerns symptoms that are observable at baseline. Moreover, baseline-symptoms PC1 scores significantly correlated with Stage 1 PC1 scores across groups (*r* = 0.20, *p =* 0.005, interaction baseline-symptoms PC1 scores × treatment: *F*_1,188_ = 0.001, *p =* 0.98), suggesting that the more severe the baseline symptomatology, the greater the improvement.

It is noteworthy that baseline-symptoms PC1 scores did not significantly differ between the sertraline and placebo groups (*t*_186_ = −0.82, *p =* 0.41), confirming that the two groups were identical at baseline.

#### CGI is not predicted by baseline clinical characteristics

In contrast, CGI response status was not predicted by any baseline characteristics (age, gender, ethnicity, education, MDD severity, MDD chronicity, HRSD, ASMR, CHRT, CAST, and baseline-symptoms PC1, all *p >* 0.08 across subjects and within each treatment group).

### Brain-behavior mapping

**Figure 4.**
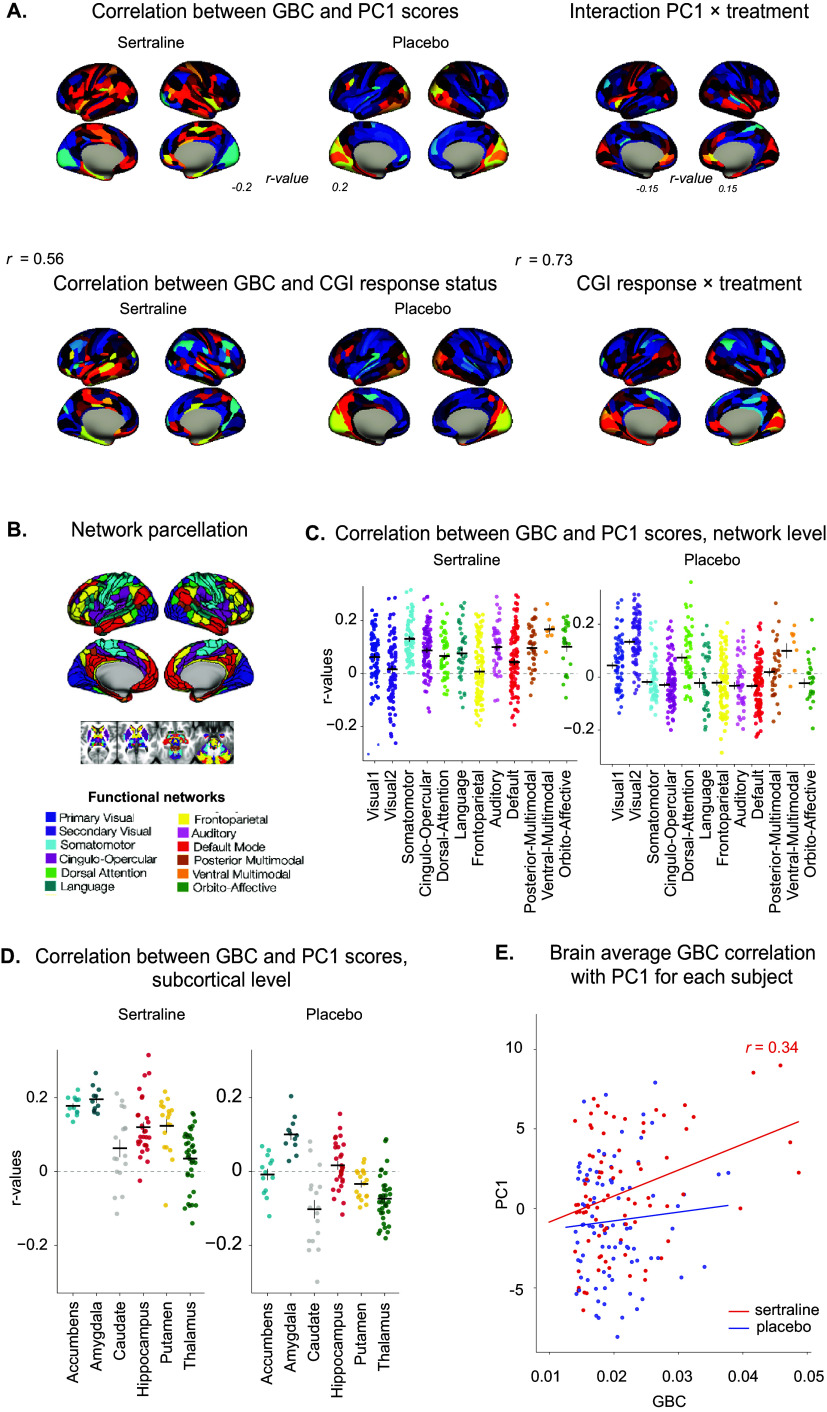
Brain–behavior mapping of mood improvement during Stage 1. (A) Top: Correlation between the parcellated resting-state GBC and the PC1 scores in the sertraline (left) and the placebo (middle) groups during Stage 1, and interaction between the two treatment groups (right). Bottom: Correlation between the parcellated resting-state GBC and the CGI response status in the sertraline (left) and the placebo (middle) groups, and interaction between the two treatment groups (right). Correlation maps are visually different between the sertraline and the placebo groups, suggesting that the baseline cerebral predictors of clinical improvement differ according to the pharmacological intervention. Exploratory analyses showed a lower correlation between GBC–PC1 brain-behavior mapping and GBC–CGI response brain-behavior map in the sertraline group compared to the placebo group, suggesting that CGI response has the same brain predictive factors as PC1 for placebo but not for sertraline. The interaction between GBC, PC1, and treatment on the one hand, and GBC, CGI response, and treatment on the other hand, displayed in the right panel, shows how each parcel contributes to the pharmacological response (as opposed to the placebo effect). (B) Network parcellation. (C) Correlation between the parcellated resting-state GBC regrouped by networks and the PC1 scores in the sertraline (left) and the placebo (right) groups. Each dot represents a parcel, and each horizontal bar represents the mean of correlation *r*-values for a given network across subjects. (D) Correlation between the parcellated resting-state GBC regrouped by subcortical regions and the PC1 scores in the sertraline (left) and the placebo (right) groups. Each dot represents a parcel, and each horizontal bar represents the mean of correlation *r*-values for a given subcortical region across subjects. (E) Brain average GBC correlation with PC1 in the two treatment groups during Stage 1 (red: sertraline and blue: placebo). Each dot represents a subject, lines represent the linear regressions, and the shaded areas represent the 95% confidence interval. PC1 scores and GBC were significantly correlated in the sertraline group (*F*_1,86_ = 11.42, *p =* 0.0011), but not in the placebo group (*F*_1,92_ = 0.59, *p* = 0.44), indicating that baseline GBC is a predictive factor of pharmacologically-induced clinical improvement.

To identify neural circuits predicting factors of response, we explored the relationship between resting-state GBC maps at baseline and clinical improvement during Stage 1. We estimated GBC calculated at the parcel level and then averaged within each predefined functional network, within each anatomically defined subcortical structure, and finally within the entire brain. To study the direction of this effect and compare GBC–PC1 relationship strength between the sertraline and the placebo groups, we computed GBC–PC1 correlation (*r*-values). The corresponding brain-behavior correlation maps and graphs are depicted in Figure 4. To capture the relationships between GBC and PC1 as a function of treatment and brain level, we then performed ANOVA following the statistical plan described in the Methods section.

#### Resting-state GBC at baseline is similar in the sertraline and the placebo groups

First, we verified that sertraline and placebo groups had a comparable brain connectivity at baseline at the parcel, network, and brain average levels and did not find any differences between the sertraline and placebo groups (all *p*_adjusted_ *>* 0.1).

#### Parcellated GBC at baseline does not significantly predict clinical improvement or response status

At the parcel level (*n* = 718, Figure 4A), the interactions between GBC and scores of common-improvement PC1 (GBC–PC1) did not survive correction for multiple comparisons (family-wise error rate correction, *α =* 0.05 in PALM (Smith & Nichols, 2009). However, GBC–PC1 brain-behavior mapping was stronger in the sertraline compared to the placebo group (*t*_717_ = 10.10, *p*_adjusted_ < 0.001).

Interestingly, there was a higher correlation between the GBC–PC1 brain-behavior map and the GBC–CGI response brain-behavior map in the placebo (*r* = 0.73, *p <* 0.001) compared to the sertraline group (*r* = 0.56, *p <* 0.001), suggesting that placebo brain-behavior mapping is less specific to the measure used for assessing clinical response.

#### Functional network GBC at baseline predicts clinical improvement in both groups

Then, we explored the brain–behavior relationships at a larger spatial scale of functional cortical networks. Using a predefined template of canonical functional networks on the same parcellation scheme, we averaged GBC across parcels belonging to each network (*n* = 12, Figure 4B).

#### GBC differs as a function of PC1 scores, and its interaction with treatment and networks

We first measured whether GBC at the network level differs as a function of PC1 across treatment groups by running an ANOVA with GBC as a dependent variable, PC1 in interaction with treatment and networks, and age, gender, and site. We observed a main effect of PC1 (*F*_1,183_ = 20.20, *p <* 0.001), and age and site (all *p* < 0.001), but no main effect of treatment group (*p* = 0.7). There was a significant interaction between PC1 and treatment (*F*_1,183_ = 10.58, *p =* 0.001), between PC1 and networks (*F*_11,2068_ = 2.26, *p =* 0.010), as well as a triple interaction between PC1, treatment, and networks (*F*_11,2068_ = 2.07, *p =* 0.020), indicating that the relationship between PC1 and GBC differed across networks and treatment groups.

#### Network-level GBC predicts PC1 scores in the sertraline group but not the placebo group

Given these results and to further explore whether network-level GBC could predict treatment outcome, we performed an ANOVA with PC1 as a dependent variable and GBC, network, age, gender, and site, separately for the sertraline and the placebo group.

In the sertraline group, there was a main effect of GBC on PC1 (*F*_1,1000_ = 6.90, *p =* 0.009) and a significant interaction between GBC and network (*F*_11,1000_ = 2.18, *p =* 0.014). Particularly, positive GBC–PC1 correlation *r*-values were observed in the somatomotor, cingulo-opercular, dorsal attention, language, auditory, posterior-multimodal, ventral-multimodal, and orbito-affective networks (all *p*_uncorrected_ < 0.04), but not in the default-mode network, fronto-parietal, and visual networks (Figure 4C). However, these network-level results did not survive Bonferroni multiple comparison corrections (*n* = 12).

In the placebo group, GBC scores did not significantly predict PC1 scores, and there was no interaction between GBC and networks (all *p >* 0.4).

#### GBC in several networks predicts PC1 scores regardless of treatment

In exploratory analyses performed for each network separately across treatment groups, GBC was predictive of response across treatment groups in the dorsal attention, the ventral-multimodal, and the two visual networks (all *F*_1,183_ > 8.00, all *p*_adjusted_ *<* 0.05).

Figure 4C depicts GBC–PC1 correlation *r*-values for each network in each treatment group to provide a sense of strength and direction of brain-behavior relationships.

#### Amygdala GBC at baseline predicts clinical improvement

Then, we averaged GBC across parcels belonging to the subcortical structure of interest, that is, amygdala, hippocampus, thalamus, and striatum with its substructures nucleus accumbens, putamen, and caudate nucleus.

#### GBC differs as a function of PC1 scores, and its interaction with treatment or subcortical structure

We first measured whether GBC at the subcortical level differs as a function of PC1, treatment groups, and subcortical structures by running an ANOVA with GBC as a dependent variable, PC1 in interaction with treatment and subcortical structures, and age, gender, and site as independent variables. We observed a main effect of PC1 (*F*_1,183_ = 10.30, *p =* 0.002), and age and site (all *p* < 0.001), but no main effect of treatment group (*p* = 0.23). There was a significant interaction between PC1 and treatment (*F*_1,183_ = 11.30, *p =* 0.001), and between PC1 and subcortical structures (*F*_11,1880_ = 2.37, *p =* 0.009). The triple interaction between PC1, treatment, and subcortical structures was not significant (*F*_11,1880_ = 0.44, *p =* 0.93).

#### Subcortical-level GBC predicts PC1 scores in the sertraline group but not the placebo group

Given these results and to further explore whether subcortical-level GBC could predict treatment outcome, we performed an ANOVA with PC1 as a dependent variable and GBC, subcortical structures, age, gender, and site, separately for the sertraline and the placebo group.

In the sertraline group, there was no main effect of GBC on PC1 (*F*_1,909_ = 0.005, *p =* 0.95), but a significant interaction between GBC and subcortical structures (*F*_11, 909_ = 2.71, *p =* 0.003). Indeed, in the sertraline group, positive *r*-values were observed in the nucleus accumbens, the amygdala, the hippocampus, and the putamen (all *p*_uncorrected_ < 0.04, see Figure 4D) but not in the caudate nucleus and thalamus. These structure-level results did not survive Bonferroni multiple comparison corrections (*n* = 6).

In contrast, in the placebo group, GBC scores did not significantly predict PC1 scores (*F*_1,969_ = 0.02, *p =* 0.90), and there was no interaction between GBC and networks (*F*_11,969_ = 0.39, *p =* 0.95).

#### Amygdala GBC predicts PC1 scores regardless of treatment

Exploratory analyses performed for each structure across treatment groups revealed that GBC in the amygdala was predictive of PC1 scores (*F*_1,183_ = 10.99, *p*_adjusted_ *=* 0.007), without interacting with treatment group.

Figure 4D depicts GBC–PC1 correlation *r*-values for each subcortical structure in each treatment group to provide a sense of strength and direction of brain-behavior relationships.

#### Whole brain averaged GBC at baseline is predictive of clinical improvement under sertraline but not under placebo

Finally, at the whole-brain level, we found that GBC averaged across 718 cortical and subcortical parcels predicted individuals’ clinical improvement (PC1 scores) in the sertraline group only. Indeed, there was a main effect of GBC and of treatment on PC1 (GBC: *F*_1,183_ = 11.72, *p <* 0.001; treatment: *F*_1,183_ = 8.74, *p =* 0.003). Although the interaction between GBC and treatment was not significant (*F*_1,183_ = 1.96, *p =* 0.16), a significant correlation between GBC and PC1 scores was observed in the sertraline group only (*r =* 0.34, *t*_91_ = 3.41, *p <* 0.001; placebo group: *r =* 0.08, *t*_97_ = 0.74, *p* = 0.46, see Figure 4E). This result suggests that baseline GBC is predictive of clinical improvement under sertraline but not under placebo.

#### Comparing brain-behavior mapping with CGI response status versus PC1 scores

To test whether GBC–PC1 brain–behavior mapping differed from GBC–CGI mapping, we performed the same analysis pipeline at the functional network and the subcortical levels with CGI response status as an independent variable instead of PC1. Overall, we observed the same pattern of results where relationships between GBC and CGI were observed in the sertraline but not in the placebo group (see Supplementary results for details). To directly compare GBC–PC1 and GBC–CGI brain-behavior mappings in each treatment group, we computed GBC–CGI correlation *r*-values and ran *t*-tests between GBC–PC1 and GBC–CGI *r*-values at the functional network and the subcortical levels. At the network level, GBC–PC1 *r*-values were significantly higher than GBC–CGI *r*-values in the sertraline group (paired *t*-test: mean of the difference: 0.53; *t*_11_ = 2.97, *p =* 0.013). This result was not observed in the placebo group (*p =* 0.23) or for subcortical structures (all *p >* 0.1).

This finding confirms what was observed at the parcel level: (1) that sertraline brain-behavior mapping differs between PC1 and CGI measure, and (2) that GBC–PC1 mapping is stronger than GBC–CGI mapping in this group.

## Discussion

### Summary of the results

We studied patterns of mood improvement in a cohort of patients with MDD from the EMBARC clinical trial, treated either with antidepressants (sertraline or bupropion) or placebo, using a data dimension reduction approach (i.e., PCA) across targeted clinical scales. This data-driven approach yielded a single dimension (PC1) that passed split-half cross-validation testing. This robust PC solution provided a low-dimensional pattern of mood improvement and was strikingly similar across both treatment and placebo, suggesting that mood improvement may evolve along a common symptom axis. Importantly, sertraline-induced PC1 scores were greater than placebo-induced PC1 scores and better predicted by baseline clinical and neural characteristics.

Such differences were not observed when using CGI to measure clinical response.

### A better characterization of mood improvement

One of the specific aspects of EMBARC is the intensity of the reported placebo response. Previous examinations of EMBARC found no differences between sertraline and placebo efficacy (Chin Fatt et al., 2020; Cooper et al., 2019; Fan et al., 2020; Pizzagalli et al., 2018; Webb et al., 2019; Whitton et al., 2019), and commonalities in predictive factors between the two groups (Cooper et al., 2019; Fan et al., 2020; Pizzagalli et al., 2018; Whitton et al., 2019; Zhao et al., 2023), supporting the idea that a placebo response is embedded into the antidepressant response (Peciña et al., 2015). In our analysis, no difference between the two groups and no clinical predictive factors could be isolated when using the CGI. However, by reducing the dimensionality of the data (PCA), we reveal a higher efficacy in the sertraline group, predicted by both clinical and neural factors. Interestingly, some patients considered as nonresponders according to the CGI and switched to another treatment had high common-improvement PC1 scores. Conversely, some patients considered as responders according to the CGI and stayed on the same treatment had low common-improvement PC1 scores. This discrepancy between routine clinical response assessment (i.e., CGI) and the low-dimensional measure of mood improvement (PC1 scores) likely stems from the broad range of symptoms captured in the PCA (Demyttenaere & De Fruyt, 2003;Maier & Philipp, 1985 ; Möller, 2001). Indeed, suicidal risk and anxiety scores significantly influenced the overall pattern in the PCA solution, especially in the sertraline group. This suggests that the efficacy of sertraline above placebo might be primarily due to its antisuicidal and anxiolytic effects. It also raises the possibility that the pure antidepressant response observed corresponds to a placebo effect. Additionally, we observed a dissociation between predictive factors and clinical improvement dimensions. In line with previous studies, baseline depression severity (HRSD scores) specifically predicted sertraline efficacy as measured by PC1 (De Carlo, Calati, & Serretti, 2016; Dodd & Berk, 2004; Webb et al., 2019), even if HRSD scores themselves were not more improved in the sertraline group compared to placebo (Chin Fatt et al., 2020; Cooper et al., 2019; Fan et al., 2020; Pizzagalli et al., 2018; Webb et al., 2019; Whitton et al., 2019).

How best to measure clinical improvement and which symptomatic dimensions should be included is a thorny question. Many clinical scales have been validated and are commonly used in research as well as in clinical practice (Beck et al., 1961; Hamilton, 1960; Montgomery & Åsberg, 1979; Rush et al., 2003; Trajković et al., 2011; Trull & Ebner-Priemer, 2009). They have strengths and weaknesses, depending on the clinical severity and subtypes of depression that should be assessed (Möller, 2001), and have therefore different fields of application (Furukawa, 2010; Nezu, McClure, & Nezu, 2015). Importantly, total scale scores can be biased by the number of items dedicated to specific domains. For example, the Hamilton Scale includes many somatic symptoms, leading to an overestimate of antidepressant efficacy for sedative medications and an underestimate for drugs associated with somatic side effects (Maier & Philipp, 1985; Möller, 2001). Similarly, whether patient or clinician assessments have higher sensitivity and specificity is a matter of debate (Bailey & Coppen, 1976; Chevance et al., 2020). Furthermore, global evaluation, such as the CGI, may be subject to a rater bias (Petkova et al., 2000). All those limitations have been extensively discussed elsewhere (Cusin, Yang, Yeung, & Fava, 2010; Demyttenaere & De Fruyt, 2003; Fried, Flake, & Robinaugh, 2022). In particular, Fried et al. (2022) highlighted that depression may be a continuum from healthy to severely depressed rather than categorical, and that depressive symptoms are multidimensional and therefore not adequately represented by the scale’s total scores. With our approach, we provide a proof-of-concept that some caveats can be circumvented by an exhaustive inclusion of multiple-scale items and their subsequent selection by data-driven reduction. Specifically, it provides a unidimensional and continuous measure based on different instruments, which may capture a more valid phenotype of symptomatic dimensions than CGI or HRSD.

### Predicting clinical improvement with baseline functional connectivity patterns

In turn, we investigated whether distinct baseline resting-state GBC patterns could predict the magnitude of PC-derived symptom improvement in each group. Interestingly, although direct correlations between GBC and PC1 did not survive multiple corrections at the parcel, network, and subcortical structure levels, we found that whole-brain averaged higher baseline GBC in the sertraline group predicted greater improvement on the PC1 score, whereas it was not the case in the placebo group. Moreover, certain brain structures’ baseline GBC, notably the amygdala, predicted clinical improvement across treatment groups. Our findings support previous analyses of the EMBARC dataset using different techniques (Chin Fatt et al., 2023; Chin Fatt, Cooper, Jha, Aslan, et al., 2021; Rolle et al., 2020), and align with studies on similar datasets (Chen et al., 2018; Godlewska et al., 2018; Karim et al., 2018; Korgaonkar et al., 2020; Li et al., 2023; Lu et al., 2020; Martens et al., 2022; Strege et al., 2023; Wu et al., 2016). However, contrary to previous findings, there was no significant predictive effect of the DMN (Chin Fatt et al., 2020, 2023; Chin Fatt, Cooper, Jha, Aslan, et al., 2021; Dichter, Gibbs, & Smoski, 2015; Goldstein-Piekarski et al., 2018) or of the rostral anterior cingulate cortex connectivity (Cooper et al., 2019; Dunlop, Talishinsky, & Liston, 2019; Kemp, Gordon, Rush, & Williams, 2008; Liston et al., 2014; Pizzagalli, 2011; Pizzagalli et al., 2018; Posner et al., 2013) on clinical improvement. Similarly, prior work shows that decreased amygdala connectivity at baseline correlates with positive clinical outcomes (Chen et al., 2008; Li et al., 2023; Liu et al., 2023; Nakamura et al., 2021; Salomons et al., 2014), whereas we, and others (Alexopoulos et al., 2012), found an opposite pattern. These mixed results may be due to the use of GBC for each parcel instead of focusing on a predefined region of interest or using connectivity measures restricted to specific networks.

### The common geometry of improvement under sertraline and placebo is underpinned by different mechanisms

More importantly, and in line with previous results (Chin Fatt et al., 2020, 2023; Chin Fatt, Cooper, Jha, Aslan, et al., 2021; Cooper et al., 2019; Zhao et al., 2023), our study shows that baseline neural patterns that relate to sertraline and placebo effects are, in fact, different. In other words, even if the pattern of mood improvement is highly similar in both groups, it seems to be related to different baseline configurations of the neural system. First, clinical improvement amplitude was greater in the sertraline group, suggesting that pharmacological action amplifies the placebo response. This result is consistent with the finding that patients under antidepressant and placebo had a similar time-course of clinical improvement but different response amplitude (Gueorguieva et al., 2011). Second, some neural and clinical factors were specifically predictive of sertraline efficacy, suggesting that pharmacological improvement relies on more reproducible neurobehavioral features with lower heterogeneity than placebo response. Additionally, in the sertraline group, brain patterns predicting clinical improvement differed according to the variable used to measure improvement (CGI vs. PC1), particularly at the network level, whereas this was not the case in the placebo group. This result has two implications. First, the direct link between CGI response status and PC1 is weaker in the sertraline group compared to the placebo group, particularly among nonresponders. This is supported by the wider distribution of PC1 scores. As the sertraline group showed a significantly greater improvement in suicidal and anxiety dimensions (both captured by PC1) compared to placebo, this suggests that CGI could be less sensitive to these specific dimensions. Second, sertraline may have different pharmacological mechanisms of action on depression and suicidal/anxiety. It could be argued that depression improvement is solely due to the placebo effect, or at least that there is a strong overlap between those two (Huneke et al., 2022). However, and crucially, even for the CGI response status, the brain maps observed in the sertraline group are very different from placebo group. This indicates that sertraline efficacy measured by CGI response status is not solely attributable to a placebo effect. Therefore, clinical improvement after sertraline seems to rely on reproducible neurobehavioral features, targeting depression and anxiety/suicidal circuits in distinct and specific ways. By contrast, the placebo effect could be noisier and less differentiated in terms of brain circuits since placebo clinical improvement was generally less predicted by brain and clinical patterns compared to sertraline. Overall, by including clinical features other than depression in the analysis, we were able to identify brain circuits that appear to be specifically affected by sertraline’s pharmacological action and go beyond placebo response.

### Limitations and perspectives

Our study has several limitations. First, it is a secondary analysis of a publicly available dataset. This analysis was, therefore, not initially planned and could have been biased by previously published data on the same cohort. Second, we did not reproduce all previously published findings, probably because we used a different analytical approach, selected a subset of participants who had clinical measures at different time points, and had a limited number of participants, considering what is needed to perform PCA, which raises the question of generalizability. In this perspective, our results would highly benefit from replication with an independent and larger dataset, particularly to explore whether the observed pattern of mood improvement is specific to this population or more universal. Third, the design did not include neuroimaging at the end of Stage 1, so we could not map clinical changes to neural evolution and could solely study predictive factors of clinical improvement. A longitudinal study would allow measuring how neural patterns evolve with time according to treatment and response. Finally, only a few conclusions could be drawn from the second stage since participants were assigned to a new treatment according to their clinical response in Stage 1, rendering the groups incomparable. Comparing different drugs in randomized parallel arms would allow examining response and predictive factors of various antidepressants, and better characterize their pharmacological mechanisms of action.

## Conclusions

In summary, we discovered a common behavioral signature of clinical improvement along the mood spectrum, with multiple symptomatic dimensions, on which patients score differently according to the treatment received. At the behavioral level, improvement under antidepressants, therefore, corresponds to an amplification of the placebo response. This improvement was more robustly predicted by baseline GBC and clinical characteristics in the sertraline group, suggesting that pharmacological improvement relies on more reproducible and specific neurobehavioral features.

## Supporting information

Berkovitch et al. supplementary material Berkovitch et al. supplementary material

## Data Availability

The dataset is publicly available on the National Institute of Mental Health Data Archive (NDA) (https://nda.nih.gov/edit_collection.html?id=2199). Codes for preprocessing can be found at the following link: https://github.com/Washington-University/HCPpipelines/pull/156. Quantitative Neuroimaging Environment & ToolboX (QuNex) was developed by our lab and is freely available at https://qunex.yale.edu/. Codes related to behavioral PCA and correlation between GBC and PC are part of N-BRIDGE (Neuro-Behavioral Relationships in Dimensional Geometric Embedding) and are described and available elsewhere (Ji et al., 2021; Lee et al., 2024; Moujaes et al., 2024). Additional custom codes are available from the corresponding author upon reasonable request.

## Supplementary material

The supplementary material for this article can be found at http://doi.org/10.1017/S0033291725100962.
