# A common symptom geometry of mood improvement under sertraline and placebo associated with distinct neural patterns – CORRIGENDUM

**DOI:** 10.1017/S003329172610422X

**Published:** 2026-04-27

**Authors:** Lucie Berkovitch, Kangjoo Lee, Jie Ji, Markus Helmer, Masih Rahmati, Jure Demsar, Aleksij Kraljic, Andraz Matkovic, Zailyn Tamayo, John Murray, Grega Repovs, John Krystal, William Martin, Clara Fonteneau, Alan Anticevic

The author would like to apologise for the missing legends and arrows from Figure 4. The correct version of Figure 4 is provided below.

**Figure 4.**
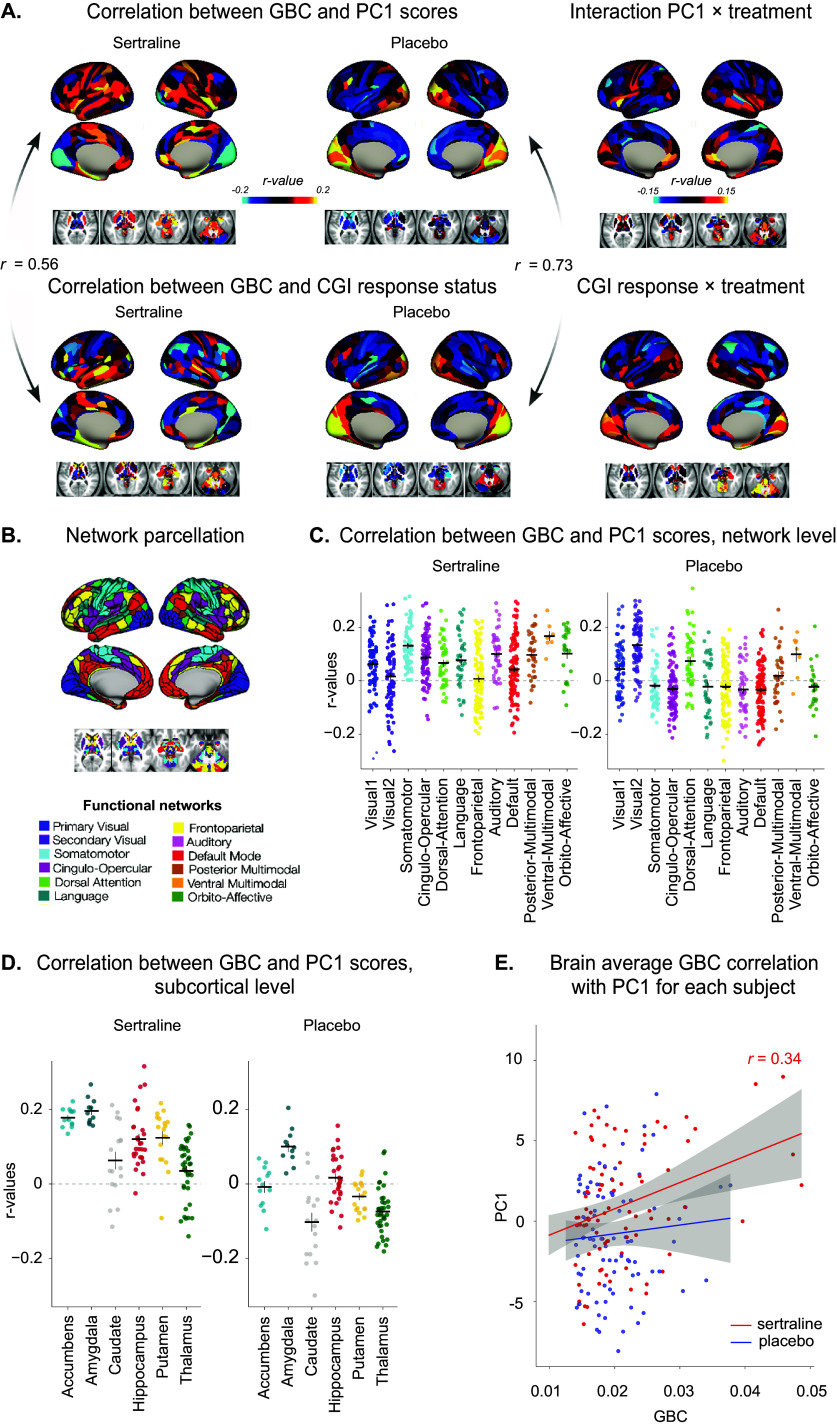
Brain–behavior mapping of mood improvement during Stage 1. (A) Top: Correlation between the parcellated resting-state GBC and the PC1 scores in the sertraline (left) and the placebo (middle) groups during Stage 1, and interaction between the two treatment groups (right). Bottom: Correlation between the parcellated resting-state GBC and the CGI response status in the sertraline (left) and the placebo (middle) groups, and interaction between the two treatment groups (right). Correlation maps are visually different between the sertraline and the placebo groups, suggesting that the baseline cerebral predictors of clinical improvement differ according to the pharmacological intervention. Exploratory analyses showed a lower correlation between GBC–PC1 brain-behavior mapping and GBC–CGI response brain-behavior map in the sertraline group compared to the placebo group, suggesting that CGI response has the same brain predictive factors as PC1 for placebo but not for sertraline. The interaction between GBC, PC1, and treatment on the one hand, and GBC, CGI response, and treatment on the other hand, displayed in the right panel, shows how each parcel contributes to the pharmacological response (as opposed to the placebo effect). (B) Network parcellation. (C) Correlation between the parcellated resting-state GBC regrouped by networks and the PC1 scores in the sertraline (left) and the placebo (right) groups. Each dot represents a parcel, and each horizontal bar represents the mean of correlation *r*-values for a given network across subjects. (D) Correlation between the parcellated resting-state GBC regrouped by subcortical regions and the PC1 scores in the sertraline (left) and the placebo (right) groups. Each dot represents a parcel, and each horizontal bar represents the mean of correlation *r*-values for a given subcortical region across subjects. (E) Brain average GBC correlation with PC1 in the two treatment groups during Stage 1 (red: sertraline and blue: placebo). Each dot represents a subject, lines represent the linear regressions, and the shaded areas represent the 95% confidence interval. PC1 scores and GBC were significantly correlated in the sertraline group (*F*_1,86_ = 11.42, *p =* 0.0011), but not in the placebo group (*F*_1,92_ = 0.59, *p* = 0.44), indicating that baseline GBC is a predictive factor of pharmacologically-induced clinical improvement.
