# Supplementary material for: A common symptom geometry of mood improvement under sertraline and placebo associated with distinct neural patterns
Source: Psychol Med. 2025 Jul 4;55:e185. doi: 10.1017/S0033291725100962 (PMC12270277; doi:10.1017/S0033291725100962)
Supplement: Berkovitch et al. supplementary material [file S0033291725100962sup001.docx]

# Supplementary methods

## Dimension reduction of symptom improvement

In step 1, we first performed data dimension reduction using the PCA of 73 items, as proposed and validated in our previous work (Ji, Diehl, et al., 2019; Lee et al., 2024; Moujaes et al., 2022). Each item was first scaled to have unit variance across patients before running the PCA. PCA was performed for subgroups defined by treatment or clinical response status (during stage 1: sertraline, placebo, responders to sertraline, non-responders to sertraline, responders to placebo, non-responders to placebo; during stage 2: switched from sertraline to bupropion, switched from placebo to sertraline, continuing sertraline, continuing placebo; all participants during stage 1, all participants during stage 2). Importantly, in the present work, we focused on capturing the principal axes of symptom improvement between two time points, by performing PCA on the difference of score for each item before and after treatment. This approach provides an overview of improvement at the item level, based on the geometry of each PC loadings. In step 2, the reproducibility of the estimated symptom PCs was evaluated using a permutation-based cross validation and null distribution based significant testing of each PC. First, significance of the derived principal components (PCs) was computed via permutation testing. For each permutation, patient order was randomly shuffled for each symptom variable before re-computing PCA. This permutation was repeated 10 000 times to establish the null model. PCs which accounted for a proportion of variance that exceeded chance (p < 0.05 across all 10 000 permutations) were retained for further analysis. PCs are ordered as a function of the behavioral variance that they account for. Second, we performed split-half permutations (1000 times) to test for reliability of the derived PCs. PC reliability was assessed through split-half correlation: only PCs with loadings correlating above 50% across 1000 permutations were considered reliable. In step 3, in order to identify whether symptom improvement shared geometry across groups, the similarity of the geometries of estimated PCs was evaluated between two groups in each comparison, using the Pearson’s correlation of the loadings of 73 behavioral measures on each PC.

## Preprocessing pipeline

Neuroimaging data were preprocessed using the Human Connectome Project (HCP) minimal preprocessing pipeline (Glasser et al., 2013), adapted for compatibility with “legacy” data, which are now featured as a standard option in the HCP pipelines provided by our team ([https://github.com/Washington-University/HCPpipelines/pull/156)](https://github.com/Washington-University/HCPpipelines/pull/156). These modifications to the HCP pipelines were necessary as the EMBARC data did not include a standard field map and did not incorporate a T2w high-resolution image or field maps. The adaptations for single-band BOLD acquisition have previously been described in detail (Ji, Spronk, et al., 2019). The adapted HCP pipeline included the following steps: (i) The T1- weighted images were corrected for bias-field distortions and warped to the standard MNI-152 brain template through a combination of linear and nonlinear transformations using the FMRIB Software Library (FSL) linear image registration tool (FLIRT) and non-linear image registration tool (FNIRT) (Jenkinson et al., 2002). (ii) FreeSurfer’s recon-all pipeline was used to compute brain-wide segmentation of gray and white matter to produce individual cortical and subcortical anatomical segmentation (Reuter et al., 2012). After completing the recon-all pipeline, all T1-weighted images were subjected to qualitative (visual) quality control (QC) by at least two independent trained researchers. (iii) Cortical surface models were generated for pial and white matter boundaries as well as segmentation masks for each subcortical gray matter voxel. Using the pial and white matter surface boundaries, a “cortical ribbon” was defined along with corresponding subcortical voxels, which were combined to generate the Connectivity Informatics Technology Initiative (CIFTI) volume/surface “gray-ordinate” space for each individual subject, which drastically reduces file management for combined surface and volume analyses and visualization and establishes a combined cortical surface and subcortical volume coordinate system (Glasser et al., 2013). (iv) The cortical surfaces were then registered to the group average HCP atlas using surface-based registration based on cortical landmark features, whereas the subcortical “volume” component of the image was brought into group atlas alignment via nonlinear registration (Glasser et al., 2013). (v) The BOLD data were motion corrected and aligned to the middle frame of every run via FLIRT. In turn, a liberal brain mask was applied to exclude signal from non-brain tissue. After initial processing in Neuroimaging Informatics Technology Initiative (NIFTI) volume space, BOLD data were converted to the CIFTI gray matter matrix by sampling from the anatomically defined gray matter cortical ribbon, whereas the subcortical voxels were isolated using subject-specific FreeSurfer segmentation. The subcortical volume component of the BOLD data was then aligned to the group atlas as part of the NIFTI processing in a single transform step that concatenates all of the transform matrices for each prior processing step (i.e., motion correction, registration, distortion correction). This produced a single nonlinear transformation to minimize interpolation cost. In turn, the cortical surface component of the CIFTI file was aligned to the HCP atlas using surface-based nonlinear deformation based on sulcal features. Following these “minimal” HCP preprocessing steps, a high-pass filter (0.008 Hz) was applied to the BOLD time series to remove low temporal frequencies and scanner drift. QuNex tools were then used to compute the signal in the ventricles, deep white matter, and across all gray matter voxels as proxy of global signal regression (GSR) to address spatially pervasive sources of artifacts (Power et al., 2017). These time series were modeled as nuisance variables and were regressed out of the gray matter voxels. Subsequent analyses used the residual BOLD time series following these denoising steps. Of note, we calculated SNR for each participant by obtaining the mean signal and SD for a given slice across the BOLD run, while excluding all non-brain voxels across all frames. In addition, we implemented “movement scrubbing,” as recommended by Power (Power et al., 2012). Movement scrubbing refers to the practice of removing BOLD volumes that have been flagged for high motion to minimize movement artifacts. Specifically, all frames with possible movement-induced artifactual fluctuations in intensity were identified via two criteria: (i) frames displacement (the sum of the displacement across all six rigid body movement correction parameters) exceeding 0.5 mm (assuming 50 mm cortical sphere radius); and (ii) the normalized root mean square (RMS) (calculated as the RMS of differences in intensity between the current and preceding frame, computed across all voxels and divided by the mean intensity) exceeding 1.6 times the median. The frames flagged by either criterion were marked for exclusion (logical or), as well as the one preceding and following the flagged frame. Subjects with >50% frames flagged were excluded from further analyses.

## Global Brain Connectivity

We computed individual parcellated neural global brain connectivity (GBC) maps (Cole et al., 2010) using a whole-brain functional network parcellation to reduce the dimensionality of the neural feature space (Ji, Spronk, et al., 2019). All data was parcellated prior to conducting functional connectivity analysis as this gave the best trade-off between the sample size needed to resolve multivariate neurobehavioral solutions and the size of the feature space. For each participant, we first computed the mean BOLD signal within each parcel. Then, the individualized resting-state functional connectivity (FC) matrix was calculated by computing the Pearson’s correlation between every parcel in the brain with all other parcels. A Fisher’s r-to-Z transform was then applied. GBC was calculated by computing every parcel’s mean FC strength with all other parcels (i.e. the mean, per row, across all columns of the FC matrix) as follows:

$$GBC\left( x \right)=\frac{1}{N}\sum_{y=1}^{N} r_{xy}$$

- where *GBC*(*x*) denotes the GBC value at parcel *x*;
- where *N* denotes the total number of parcels;
- where X denotes the sum from *y* =1 to *y* = *N*;
- where *r_xy_* denotes the correlation between the time-series of parcels *x* and *y*;

GBC is a data-driven summary measure of connectedness that is unbiased with regards to the location of a possible alteration in connectivity (Cole et al., 2016) and is therefore a principled way for reducing the number of neural features while assessing neural variation across the entire brain.

# Supplementary results

## Analysis of total scale scores

In this section, we describe total scale scores for the HRSD, ASRM, CHRT and CASTS at different time points (baseline, 8 weeks, 16 weeks) or their evolution during stage 1 and 2 (for stage 1: baseline minus 8 weeks, and for stage 2: 8 weeks minus 16 weeks).

### Stage 1

Patients in the placebo and the sertraline groups were similar at baseline (see Table 1 in main text), as can be observed by superimposing their symptomatology item-by-item (see Figure 2A in the main text). After 8 weeks, the proportion of responders and non-responders according to the CGI did not significantly differ between the placebo and the sertraline groups (placebo 39.4% vs. sertraline 51.6%, *χ*^2^ = 2.4, *p* = 0.12). When computing the difference between 8 weeks and baseline mean scores, there was a higher improvement in the sertraline compared to the placebo group for the CHRT scores (CHRT propensity score improvement: sertraline: mean: 11.3 (SD: 8.73) vs. placebo: 7.8 (9.53), *t*_190_ = 2.63, *p* = 0.009; CHRT risk score improvement: sertraline: 2.30 (2.49) vs. placebo: 1.12 (2.75), *t*_190_ = 3.12, *p* = 0.002) but not for the other scales (HRSD: *t*_189_ = 1.20, *p* = 0.23; ASRM: *t*_180_ = -1.48, *p* = 0.14 and CAST: *t*_190_ = 1.74, *p* = 0.08). Noteworthy, the insomnia subscore evolution of the CAST (Trombello et al., 2018) was larger in the sertraline group, which may reflect a side effect of the treatment (insomnia: mean sertraline: 2.26 vs. placebo: 0.097, *t*_190_ = 3.61, *p* < 0.001; irritability: *t*_189_ = 1.35, *p* = 0.18; anxiety: *t*_179_ = 0.06, *p* = 0.95; panic: *t*_186_ = -0.74, *p* = 0.46).

When splitting the participants in each treatment group according to the clinical response status (responders vs. non-responders according to the CGI at 8 weeks), we observed significant differences in symptom improvement for all total scale scores (sertraline group: all *p* < 0.007; placebo group: all *p* < 0.02, see Figure 2C in main text). However, there was no significant difference in baseline demographics or total scale scores between responders and non-responders (age, gender, ethnicity, education, MDD severity, MDD chronicity, HRSD, ASMR, CHRT propensity, CHRT risk, CAST, all *p* > 0.08 across subjects and within each treatment group), suggesting that baseline symptoms are not predictive of subsequent clinical response status.

### Stage 2

Between week 8 and week 16, participants’ medication depended on clinical response status at the end of stage 1: non-responders to sertraline were switched to bupropion, non-responders to placebo were switched to sertraline and responders kept their treatment the same (see Figure 1 in main text).

At the end of stage 1, non-responders to sertraline had lower CHRT risk score than non-responders to placebo (sertraline switch to bupropion: 3.79 (2.35) vs. placebo switch to sertraline: 5.06 (2.54), *t*_75_ = 2.34, *p* = 0.022, see Figure 2D). During stage 2, patients introduced with sertraline improved more than patients switched to bupropion for their CHRT propensity scores (sertraline (previously placebo): 8.16 (10.0) vs. bupropion (previously sertraline): 3.47 (6.1), *t*_80_ = 2.65, *p* = 0.010), CHRT risk scores (sertraline: 2.0 (2.27) vs. bupropion: -0.1 (2.28), *t*_71_ = 4.01, *p* = 0.0002), and CAST scores (sertraline: 5.76 (9.27) vs. bupropion: 1.88 (7.72), *t*_78_ = 2.07, *p* = 0.040). Responders to sertraline and placebo did not significantly differ in their total scale scores at the end of stage 1 (all *p* > 0.1) and did not significantly change during stage 2 (all *p* > 0.06), nor differ in their improvements (all *p* > 0.05).

## Shared PC geometry

Eighteen items had positive loadings above the 3rd quartile for the common PC1 of stage 1. Eight items of the HRSD: depressed mood, work and activities, worthlessness, hopelessness, helplessness, concentration, social withdrawal, fatigability; 5 items of the CHRT: I can do nothing right, everything I do turns out wrong, things will never get better, I have thoughts about how I might kill myself, no future; 4 items of the CAST: Lately everything seems to be annoying to me, I find people get on my nerves easily, I wish to be alone, anxiety. One item of the CAST had a negative loading under the 3rd quartile across all PC1: feeling good (see supplementary Figure S3).


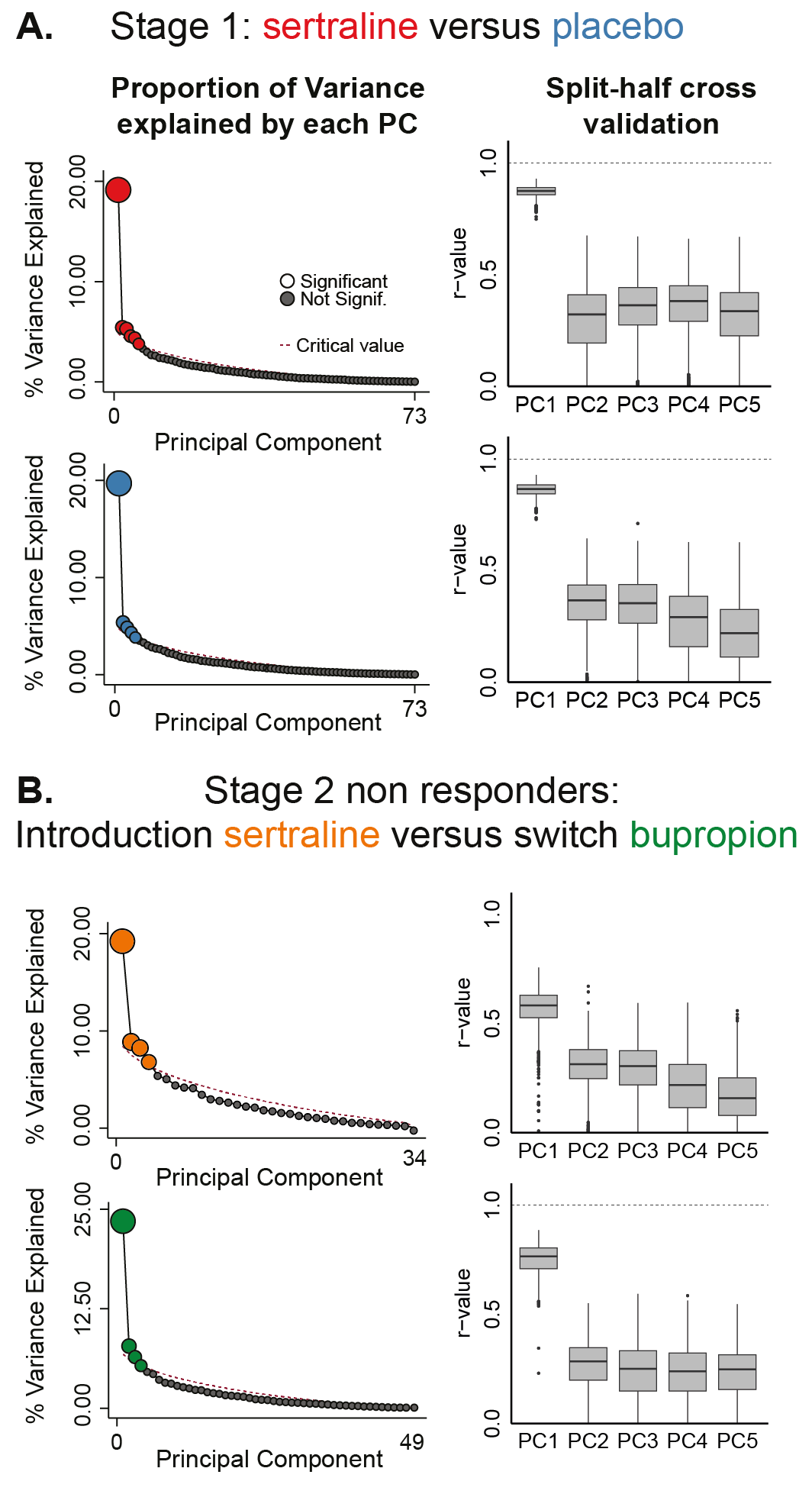


**Supplementary Figure S1.** Proportion of variance explained by PC in each group and split-half cross validation (**A.** stage 1, top: sertraline, bottom: placebo; **B.** stage 2, top: sertraline, bottom: bupropion). Only PC1, which explains the most variance, was reliable (*r*-value > 0.5) and therefore retained for further analyses.


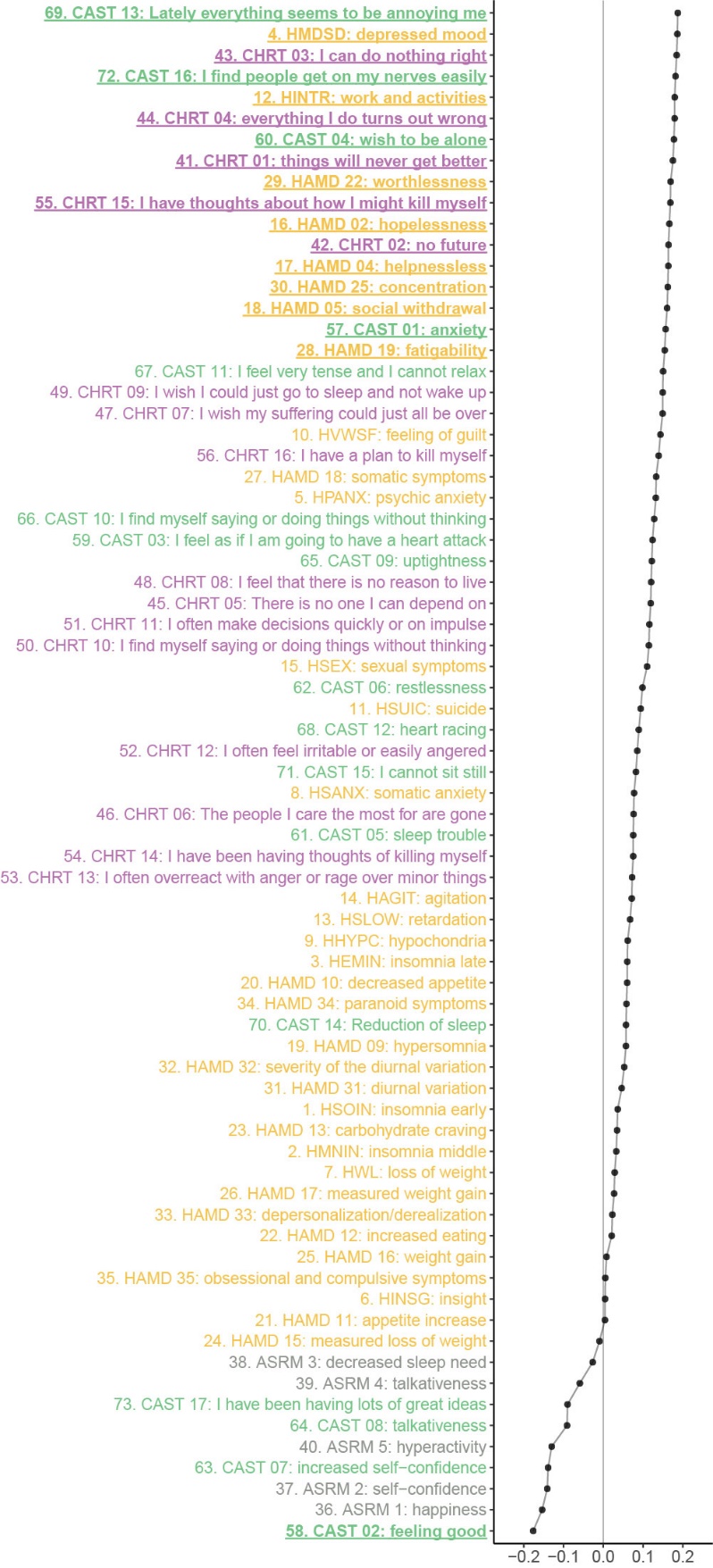


**Supplementary Figure S2. Loadings of common PC1 during stage 1.** The items above the 3rd quartile in absolute value are underlined.

## HRSD vs. CGI response mapping onto PC1

Response is usually measured as a 50% reduction in HRSD. To ensure that this metric was comparable to CGI response status in this dataset, we compared HRSD response, as defined by a 50% reduction of symptoms, and CGI response status. We found that HRSD response and CGI response status were highly superimposable (*χ*^2^ = 118.32, *p* < 0.001). Importantly, they also mapped similarly onto PC1 as depicted in the supplementary Figure S3. These results suggest that CGI appropriately reflects HRSD evolution. Noteworthy, there was an overlap for PC1 scores between responders and non-responders that seems to be independent from the method used to determine response status (CGI or 50% reduction of baseline HRSD scores). This result indicates that the mismatch between PC1 scores and response status may be driven by scales items other than HRSD. In this perspective, PC1 scores likely reflects a more complex and granular symptom measure of clinical improvement that is not fully accounted for by a binary response status based on a single clinical scale.


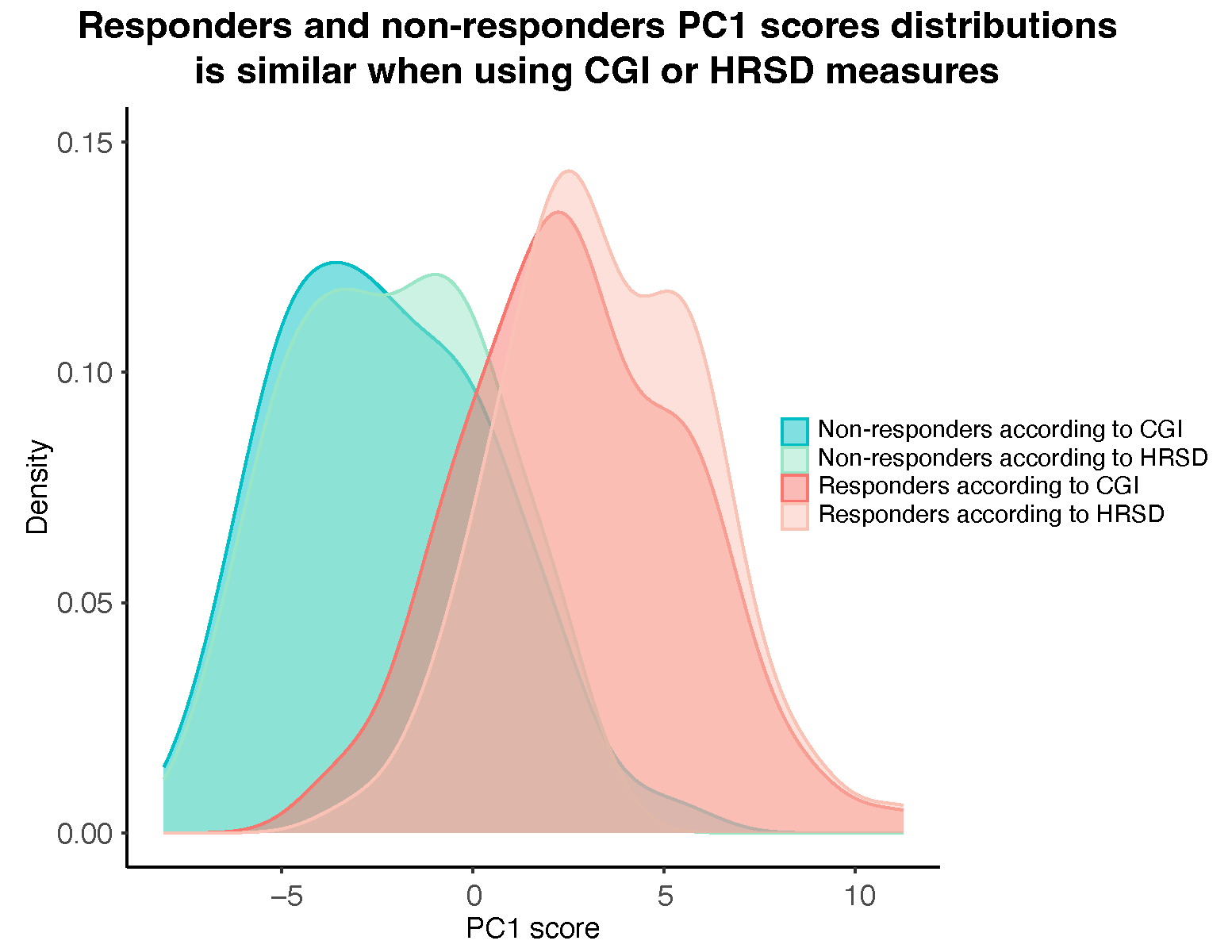


**Supplementary Figure S3.** PC1 scores distributions density is highly similar between responders according to CGI and HRSD (dark pink and light pink, respectively) and non-responders according to CGI and HRSD (dark cyan and light cyan, respectively).

## Brain-behavior mapping for CGI response status

At the parcel level, the interactions between GBC and the CGI response status (GBC-CGI response) did not survive correction for multiple comparisons (family-wise error rate correction, *α* = 0.05 in PALM (Smith & Nichols, 2009)).

At the network level, ANOVA with GBC as a dependent variable, CGI in interaction with treatment and network; age, gender and site as independent variables revealed a significant main effect of CGI response (*F*_1_*_,_*_183_ = 10.03, *p* = 0.002), an interaction between CGI and treatment (*F*_1_*_,_*_183_ = 7.61, *p* = 0.006), and between CGI and networks (*F*_11_*_,_*_2068_ = 2.12, *p* = 0.017). The triple interaction between CGI, treatment and networks was not significant. When splitting groups and running an ANOVA with CGI as a dependent variable and GBC, age, gender and site, yielded a significant interaction between GBC and networks in the sertraline group (*F*_11_*_,_*_1000_ = 2.65, *p* = 0.002) and no significant effect in the placebo group (all *p* > 0.8). Positive GBC-CGI *r*-values were observed in the sertraline group for the auditory, ventral-multimodal and orbito-affective networks (all *p_uncorrected_* < 0.04), but these results did not survive multiple comparison correction.

At the subcortical level, in the ANOVA with GBC as a dependent variable, CGI in interaction with treatment and subcortical structures, age, gender and site as independent variables, the only significant effect involving CGI was the interaction between CGI and treatment (*F*_1_*_,_*_183_ = 8.40, *p* = 0.004). When splitting groups and running an ANOVA with CGI as a dependent variable and GBC, age, gender and site, no significant effect was observed (all *p* > 0.1).

Finally, at the whole-brain level, GBC was significantly predictive of CGI (*F*_1_*_,_*_183_ = 4.85, *p* = 0.029) but there was no main effect of treatment on CGI (*F*_1_*_,_*_183_ = 2.29, *p* = 0.13), or interaction between GBC and treatment (*F*_1_*_,_*_183_ = 0.87, *p* = 0.35). A significant correlation between GBC and CGI was observed in the sertraline group (*r* = 0.23, *t_91_* = 2.22, *p* = 0.029), but not in the placebo group (*r* = 0.05, *t_97_* = 0.44, *p* = 0.66).

# References

Cole, M. W., Pathak, S., & Schneider, W. (2010). Identifying the brain’s most globally connected regions. *NeuroImage*, *49*(4), 3132‑3148. https://doi.org/10.1016/j.neuroimage.2009.11.001

Cole, M. W., Yang, G. J., Murray, J. D., Repovš, G., & Anticevic, A. (2016). Functional connectivity change as shared signal dynamics. *Journal of Neuroscience Methods*, *259*, 22‑39. https://doi.org/10.1016/j.jneumeth.2015.11.011

Glasser, M. F., Sotiropoulos, S. N., Wilson, J. A., Coalson, T. S., Fischl, B., Andersson, J. L., Xu, J., Jbabdi, S., Webster, M., Polimeni, J. R., Van Essen, D. C., Jenkinson, M., & WU-Minn HCP Consortium. (2013). The minimal preprocessing pipelines for the Human Connectome Project. *NeuroImage*, *80*, 105‑124. https://doi.org/10.1016/j.neuroimage.2013.04.127

Jenkinson, M., Bannister, P., Brady, M., & Smith, S. (2002). Improved optimization for the robust and accurate linear registration and motion correction of brain images. *NeuroImage*, *17*(2), 825‑841. https://doi.org/10.1016/s1053-8119(02)91132-8

Ji, J. L., Diehl, C., Schleifer, C., Tamminga, C. A., Keshavan, M. S., Sweeney, J. A., Clementz, B. A., Hill, S. K., Pearlson, G., Yang, G., Creatura, G., Krystal, J. H., Repovs, G., Murray, J., Winkler, A., & Anticevic, A. (2019). Schizophrenia Exhibits Bi-directional Brain-Wide Alterations in Cortico-Striato-Cerebellar Circuits. *Cerebral Cortex (New York, N.Y.: 1991)*, *29*(11), 4463‑4487. https://doi.org/10.1093/cercor/bhy306

Ji, J. L., Spronk, M., Kulkarni, K., Repovš, G., Anticevic, A., & Cole, M. W. (2019). Mapping the human brain’s cortical-subcortical functional network organization. *NeuroImage*, *185*, 35‑57. https://doi.org/10.1016/j.neuroimage.2018.10.006

Lee, K., Ji, J. L., Fonteneau, C., Berkovitch, L., Rahmati, M., Pan, L., Repovš, G., Krystal, J. H., Murray, J. D., & Anticevic, A. (2024). Human brain state dynamics are highly reproducible and associated with neural and behavioral features. *PLOS Biology*, *22*(9), e3002808. https://doi.org/10.1371/journal.pbio.3002808

Moujaes, F., Ji, J. L., Rahmati, M., Burt, J., Schleifer, C. H., Adkinson, B., Savič, A., Santamauro, N., Tamayo, Z., Diehl, C., Kolobaric, A., Flynn, M., Rieser, N. M., Fonteneau, C., Camarro, T., Xu, J., Cho, Y. T., Repovš, G., Fineberg, S. K., … Anticevic, A. (2022). *Ketamine induces multiple individually distinct whole-brain functional connectivity signatures* [Preprint]. Neuroscience. https://doi.org/10.1101/2022.11.01.514692

Power, J. D., Barnes, K. A., Snyder, A. Z., Schlaggar, B. L., & Petersen, S. E. (2012). Spurious but systematic correlations in functional connectivity MRI networks arise from subject motion. *NeuroImage*, *59*(3), 2142‑2154. https://doi.org/10.1016/j.neuroimage.2011.10.018

Power, J. D., Plitt, M., Laumann, T. O., & Martin, A. (2017). Sources and implications of whole-brain fMRI signals in humans. *NeuroImage*, *146*, 609‑625. https://doi.org/10.1016/j.neuroimage.2016.09.038

Reuter, M., Schmansky, N. J., Rosas, H. D., & Fischl, B. (2012). Within-subject template estimation for unbiased longitudinal image analysis. *NeuroImage*, *61*(4), 1402‑1418. https://doi.org/10.1016/j.neuroimage.2012.02.084

Trombello, J. M., Killian, M. O., Liao, A., Sanchez, K., Greer, T. L., Walker, R., Grannemann, B., Rethorst, C. D., Carmody, T., & Trivedi, M. H. (2018). Psychometrics of the self-report Concise Associated Symptoms Tracking Scale (CAST-SR) : Results from the STRIDE (CTN-0037) study. *The Journal of clinical psychiatry*, *79*(2), 17m11707. https://doi.org/10.4088/JCP.17m11707
